# Clinico-Epidemiological Study of Facial Dermatoses

**DOI:** 10.7759/cureus.99976

**Published:** 2025-12-23

**Authors:** Mansi Tiwari, Meenakshi Tripathi, Anshul Tiwari, Pooja Thagele, Nidhi Choudhary, Nidhi Rana

**Affiliations:** 1 Department of Dermatology, Ram Krishna Dharmarth Foundation (RKDF) Medical College Hospital and Research Centre, Bhopal, IND

**Keywords:** acne vulgaris, clinico-epidemiological, cross-sectional study, facial dermatoses, melasma, pigmentation disorders, tinea faciei

## Abstract

Background: Facial dermatoses constitute a diverse group of skin conditions that can significantly impact patients' quality of life due to the cosmetic and psychological implications. Understanding the clinico-epidemiological patterns of these conditions aids in timely diagnosis and effective management.

Objective: This study aims to determine the clinical and epidemiological profile of patients presenting with facial dermatoses in a tertiary care dermatology outpatient setting.

Materials and methods: A descriptive cross-sectional study was conducted in the Department of Dermatology, Venereology, and Leprology of a tertiary care hospital over 18 months (April 2023 to September 2024). Four hundred patients (n=400) of all ages and both sexes presenting with facial dermatoses were enrolled consecutively. A structured pro forma was used to collect demographic and clinical data. Relevant investigations, such as potassium hydroxide mount, Wood's lamp examination, and skin biopsy, were performed as indicated. Data were analyzed using Epi Info v3.4.5 and 7.1.5 (U.S. Centers for Disease Control and Prevention, Atlanta, GA, USA).

Results: Out of 400 patients, 242 (60.5%) were females and 158 (39.5%) were males. The most affected age group was 21-30 years, comprising 114 (28.5%) patients. Pigmentary disorders were the most common category, observed in 123 (30.8%) patients, with melasma being the leading condition, affecting 82 (20.5%) patients. Infectious dermatoses were seen in 95 (23.8%) cases, with tinea faciei being the most frequent, affecting 42 (10.5%) patients. Acne vulgaris was present in 65 (16.3%) patients, and eczema in 48 (12%) patients. A significant association was found between pigmentary dermatoses and female gender (p<0.05). Seasonal variation and history of prior treatment were noted in a considerable number of patients, accounting for 176 (44%) and 139 (34.8%) cases, respectively.

Conclusions: Facial dermatoses are more prevalent in young adults and females, with pigmentary disorders, infections, and acne being the most frequent presentations. The findings underscore the importance of public awareness, early intervention, and further research to investigate risk factors and develop effective preventive strategies.

## Introduction

The face is the most visible part of the human body and plays a central role in personal identity, social interaction, and perception [1]. As a result, dermatological conditions involving the facial region often lead to significant psychological distress, social embarrassment, and impairment in quality of life, even when the condition may be clinically mild. Facial dermatoses encompass a broad range of skin disorders, including infectious, inflammatory, autoimmune, allergic, neoplastic, and idiopathic conditions [2]. The spectrum and prevalence of these dermatoses vary widely depending on factors such as geographic region, environmental exposures, age, sex, hygiene practices, and genetic predisposition [2,3].

In India, with its diverse climate, wide-ranging socioeconomic conditions, and varied cultural practices, the pattern of facial dermatoses can present unique epidemiological trends [4]. However, there remains a relative paucity of comprehensive clinic-based studies that explore the epidemiology and clinical characteristics of facial dermatoses in the Indian context, particularly in tertiary care settings [5,6]. Understanding these patterns is crucial not only for early diagnosis and appropriate management but also for planning preventive dermatological services and awareness programs.

To the best of our knowledge, this is one of the first clinic-based studies from Central India focusing exclusively on the clinico-epidemiological patterns of facial dermatoses. This study aims to bridge the gap by conducting a detailed clinico-epidemiological assessment of patients presenting with facial dermatoses at the Department of Dermatology, Venereology, and Leprology of a tertiary care center. By analyzing demographic data, clinical presentations, potential risk factors, and diagnostic modalities, the study aims to provide valuable insights into the burden and characteristics of facial dermatoses, thereby enabling clinicians to make more informed diagnoses, manage patients more effectively, and offer better counseling.

## Materials and methods

A descriptive cross-sectional study was conducted to assess the clinico-epidemiological profile of patients presenting with facial dermatoses. The study was conducted in the Outpatient Department (OPD) of Dermatology, Venereology, and Leprology at a tertiary care center from April 2023 to September 2024, spanning 18 months.

The study population included all patients presenting to the dermatology OPD with complaints of skin lesions localized to the facial region, irrespective of age and sex. Patients of all age groups and sexes presenting with facial dermatoses and those who provided written informed consent to participate in the study were included. Additionally, patients or their guardians (in the case of minors) provided written informed consent to participate in the study.

The sample size was calculated using the standard formula. Applying this formula, the calculated sample size was 384, which was rounded off to 400 patients. A consecutive sampling technique was used. All eligible patients presenting during the study period who met the inclusion criteria were enrolled until the desired sample size was reached.

Data were collected using a structured, pre-tested pro forma that captured comprehensive information across several domains. Demographic details included age, sex, place of residence (urban or rural), and occupation. Clinical history focused on the onset, duration, and progression of facial lesions, along with associated symptoms such as itching, burning, and scaling. It also addressed recurrence patterns, seasonal variation, and any family history of similar conditions. Medical history was documented, particularly noting comorbidities such as diabetes, hypothyroidism, and polycystic ovarian syndrome (PCOS). Treatment history included details of any prior medications or dermatological procedures. Additionally, all patients underwent a comprehensive physical examination and a detailed dermatological evaluation, focusing on the morphology, distribution, extent, and symmetry of facial lesions, as well as the involvement of other body sites. The psychosocial impact was assessed through informal clinician judgment and patient self-report during the history-taking process. No validated psychometric instrument was employed.

Relevant investigations were carried out based on clinical indications. A 10% potassium hydroxide (KOH) mount was performed in cases of suspected fungal infections to detect the presence of fungal hyphae. Wood’s lamp examination was utilized in pigmentary disorders to evaluate the depth and nature of pigmentation. In selected cases with atypical, persistent, or suspected autoimmune lesions, a skin biopsy was conducted to obtain histopathological confirmation and aid in accurate diagnosis.

Statistical analysis

Data were manually coded at the time of collection and subsequently entered into Microsoft Excel (Microsoft Corp., Redmond, WA, USA) for tabulation and initial processing. Statistical analysis was performed using Epi Info versions 3.4.5 and 7.1.5 (U.S. Centers for Disease Control and Prevention, Atlanta, GA, USA). Descriptive statistics (mean, standard deviation, percentages, and proportions) were used to summarize demographic and clinical data. The chi-square test was used to find associations between categorical variables (e.g., gender vs. type of dermatosis). A p-value < 0.05 was considered statistically significant.

Ethical considerations

The study was initiated after obtaining ethical clearance from the Institutional Ethics Committee of RKDF Medical College and Research Centre, dated 1st May 2023, with registration number IECRKDFMCRC/32/2023. All participants or their guardians (in the case of minors) were enrolled after obtaining written informed consent. Written consent was also obtained for the publication of clinical photographs. Confidentiality and anonymity of patient information were strictly maintained throughout the study.

## Results

Out of the 400 patients, 226 (56.5%) were females and 174 (43.5%) were males. The age of patients ranged from 1 year to 78 years, with a mean age of 29.4 ± 14.8 years. The highest number of cases was observed in the 21-30 year age group (128 (32%)), followed by the 11-20 year age group (100 (25%)) (Table 1).

**Table 1:** Age and gender distribution of patients Data is presented as the number of patients (percentage).

Age group (years)	Male (n=174)	Female (n=226)	Total (%)
0-10	10 (5.7%)	16 (7.1%)	26 (6.5%)
11-20	44 (25.3%)	56 (24.8%)	100 (25%)
21-30	60 (34.5%)	68 (30.1%)	128 (32%)
31-40	28 (16.1%)	36 (15.9%)	64 (16%)
41-50	14 (8.0%)	26 (11.5%)	40 (10%)
51-60	12 (6.9%)	16 (7.1%)	28 (7%)
>60	6 (3.5%)	8 (3.5%)	14 (3.5%)

**Figure 1:**
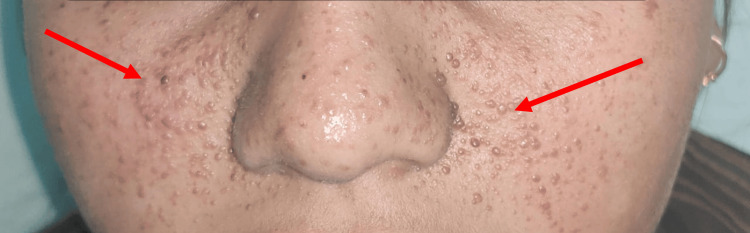
Clinical picture of a patient suffering from facial angiofibromas (red arrows)

**Figure 2:**
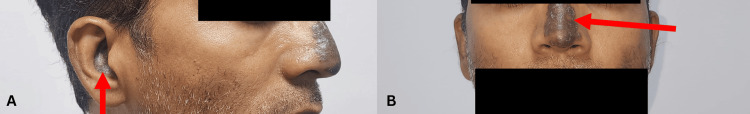
Cutaneous lupus erythematosus presenting as a hyperpigmented, scaly, and pruritic plaque on the inner ear (A) and over the nose (B), present for six months

The majority of patients (245, 61.25%) belonged to urban areas, while 155 (38.75%) were from rural areas. The most commonly diagnosed facial dermatoses were acne vulgaris (152 (38%)), followed by melasma (56 (14%)), seborrheic dermatitis (48 (12%)), and tinea faciei (36 (9%)). Less common conditions included rosacea, lupus erythematosus, contact dermatitis, and vitiligo (Table 2). Figure 1 and Figure 2 show the clinical images of the patients.

**Table 2:** Distribution of facial dermatoses

Diagnosis	Frequency (n=400)	Percentage (%)
Acne vulgaris	152	38.0
Melasma	56	14.0
Seborrheic dermatitis	48	12.0
Tinea faciei	36	9.0
Contact dermatitis	28	7.0
Vitiligo	24	6.0
Rosacea	18	4.5
Lupus erythematosus	12	3.0
Perioral dermatitis	10	2.5
Miscellaneous	16	4.0

About 160 (40%) of patients reported symptoms lasting more than six months, while 112 (28%) presented within one month of onset. Chronicity was more associated with pigmentary and autoimmune dermatoses.

A positive family history of similar facial dermatoses was seen in 76 (19%) cases, with acne being the most common. Recurrent episodes were observed in 112 (28%) patients, more commonly associated with seborrheic dermatitis and tinea faciei.

Out of 400 patients, KOH mounts were performed in 96 suspected fungal cases, yielding positive results in 78 (81.3%). Wood's lamp examination was conducted in 112 cases of pigmentary disorders; enhancement of pigmentation was noted in 42 (37.5%). Skin biopsy was required in 24 patients, confirming diagnoses such as lupus erythematosus, discoid lupus, and sarcoidosis.

A total of 86 patients (21.5%) had comorbid conditions. The most common were PCOS in females with acne (22 cases), diabetes mellitus in patients with tinea faciei (12 cases), and hypothyroidism in melasma and vitiligo (18 cases).

Table 3 shows that acne vulgaris is the most common facial dermatosis in both genders, while melasma predominantly affects females. Males had a higher proportion of varied conditions classified under "others."

**Table 3:** Gender-wise distribution of select dermatoses Data are presented as the number of patients (percentage). A p-value of <0.05 is considered significant. Others include rosacea, lupus erythematosus, contact dermatitis, perioral dermatitis, and miscellaneous dermatoses. χ²: chi-square value, df: degree of freedom

Diagnosis	Male (n = 174)	Female (n = 226)	Total (n = 400)	χ²/df/effect size (Cramér's V)	p-value
Acne vulgaris	58 (33.3%)	94 (41.6%)	152 (38.0%)	1.365/5/0.058	0.689
Melasma	6 (3.4%)	50 (22.1%)	56 (14.0%)		
Seborrheic dermatitis	22 (12.6%)	26 (11.5%)	48 (12.0%)		
Tinea faciei	20 (11.5%)	16 (7.1%)	36 (9.0%)		
Vitiligo	12 (6.9%)	12 (5.3%)	24 (6.0%)		
Others*	56 (32.2%)	28 (12.4%)	84 (21.0%)		
Total	174 (100%)	226 (100%)	400 (100%)		

A trend of increased fungal dermatoses during monsoon months and aggravation of melasma and lupus during summer was noted. Acne vulgaris showed no significant seasonal variation (p=0.882).

Out of the 400 participants, 74 (18.5%) reported a moderate to severe psychosocial impact, especially those with melasma, vitiligo, and acne scarring. Patients expressed concerns regarding appearance, marriage prospects, and workplace discrimination.

## Discussion

Facial dermatoses encompass a diverse array of skin conditions that significantly impact patients' quality of life due to their prominent visibility. This study aimed to delineate the clinico-epidemiological profile of facial dermatoses in patients attending a tertiary care center and to compare these findings with those in the existing literature.

In our study, the majority of patients were females (56.5%), with the highest prevalence observed in the 21-30-year age group (32%). This aligns with findings by Jain and Khandpur [5], who reported a female-to-male ratio of 1.2:1 and a peak incidence in the third decade of life. Similarly, Ravindranath et al. [7] observed a female preponderance (62.9%) among adolescents with facial dermatoses. The predominance in females may be attributed to hormonal influences and greater cosmetic concerns, leading to increased healthcare-seeking behavior.

Environmental pollution, particularly in urban areas, plays a significant role in the pathogenesis and exacerbation of facial dermatoses. Airborne pollutants such as particulate matter (PM2.5, PM10), nitrogen dioxide, and polycyclic aromatic hydrocarbons can penetrate the skin barrier, induce oxidative stress, and trigger inflammatory cascades. These effects can exacerbate conditions such as acne, melasma, and seborrheic dermatitis by disrupting sebum production, altering the skin microbiota, and promoting melanogenesis. Studies have shown a correlation between high pollution levels and increased severity of acne and pigmentary disorders, especially in urban populations. Hence, pollution may act as both a direct irritant and an indirect aggravating factor in facial dermatoses.

The most common facial dermatosis identified was acne vulgaris, followed by melasma, seborrheic dermatitis, and tinea faciei. The high prevalence of acne is consistent with studies by Ravindranath et al. [7], who reported acne vulgaris as the most common dermatosis (34.8%) among adolescents. However, Bhagwat et al. [8] found infections to be the most prevalent (37%), with tinea faciei comprising 18% of cases, which is higher than the 9% observed in our study. This discrepancy may be due to regional variations in climate and hygiene practices.

Melasma was the predominant pigmentary disorder in our study, accounting for 14% of cases. This is lower than the 26.7% reported by Charles et al. [9] and the 71% reported by Chintada et al. [10]. The variation could be due to differences in study populations, geographic locations, and sun exposure levels. Gupta and Mahajan [11] focused exclusively on male patients with facial hypermelanosis and found melasma in 76.7% of cases, highlighting a significant burden in this subgroup.

Tinea faciei was diagnosed in 9% of our patients, which is lower than the 18% reported by Bhagwat et al. [8]. This variation may be attributed to differences in environmental factors, personal hygiene practices, and the prevalence of fungal infections in different regions.

We observed that 21.5% of patients had comorbid conditions, with PCOS being notably associated with acne in females. This association highlights the importance of a multidisciplinary approach in managing facial dermatoses, taking into account underlying systemic conditions.

We noted an increase in fungal infections during monsoon months and an aggravation of melasma and lupus erythematosus during summer. These findings suggest that environmental factors play a role in exacerbating certain facial dermatoses, emphasizing the need for season-specific preventive measures.

A significant proportion of patients (18.5%) reported moderate to severe psychosocial impact due to their facial dermatoses. This highlights the necessity for psychological support and counseling as integral components of dermatological care.

Our study revealed a significantly higher prevalence of melasma in females (22.1%) compared to males (3.4%), consistent with Sarkar et al. [12], who reported a female-to-male ratio of 4:1. This trend, along with the predominance of acne vulgaris in females, underscores the influence of gender on facial dermatoses. Factors such as hormonal fluctuations, cosmetic use, and greater healthcare-seeking behavior likely contribute to the higher incidence in women. In contrast, the wider variety of dermatoses seen in males may be linked to greater outdoor exposure, occupational risk factors, and a tendency to seek care only for more severe conditions [13-15]. These findings underscore the importance of gender-sensitive approaches in the prevention, diagnosis, and management of facial skin disorders.

Limitations

Our study's cross-sectional design limits causal inference, and being single-center in Central India, the findings may not be generalizable nationwide due to regional variations in environment, culture, and occupation. The absence of multivariate analysis restricts the identification of independent predictors, and seasonal trends were not fully explored due to a lack of detailed statistical comparisons or time-series analysis.

## Conclusions

This study provides a comprehensive overview of the clinico-epidemiological patterns of facial dermatoses in a tertiary care setting. The findings highlight the prevalence of acne vulgaris and melasma, with notable gender differences and significant psychosocial implications. Comparisons with existing literature reveal regional and demographic variations, underscoring the need for tailored approaches in managing facial dermatoses. Further multicenter studies are warranted to understand these conditions better and develop effective prevention and treatment strategies.
